# A Better Way to Decrease Knee Swelling in Patients with Knee Osteoarthritis: A Single-Blind Randomised Controlled Trial

**DOI:** 10.1155/2019/8514808

**Published:** 2019-05-02

**Authors:** Zübeyir Sari, Onur Aydoğdu, İlkşan Demirbüken, S. Ufuk Yurdalan, M. Gülden Polat

**Affiliations:** Marmara University, Faculty of Health Sciences, Department of Physiotherapy and Rehabilitation, Istanbul, Turkey

## Abstract

**Objective:**

In this study, we compared the effects of intermittent pneumatic compression along with conventional treatment with cold-pack treatment along with conventional treatment on clinical outcomes in patients with knee osteoarthritis.

**Methods:**

Eighty-nine patients with knee osteoarthritis participated in this study. One group received ultrasound, transcutaneous electrical nerve stimulation, electrical stimulation, exercise, and cold packs. The second group received ultrasound, transcutaneous electrical nerve stimulation, electrical stimulation, exercise, and intermittent pneumatic compression. Range of motion, muscle strength, knee swelling, pain intensity, and functional status were measured at baseline and 4th week.

**Results:**

We found significant improvements in range of motion, muscle strength, pain intensity, and functional status after the treatment in both groups (*p* < 0.05). When comparing the effects of these two treatment programs, it was observed that the intermittent pneumatic compression treatment group had a better outcome in terms of knee swelling (*p*=0.028).

**Conclusions:**

According to the results, we could report that intermittent pneumatic compression therapy in addition to conventional treatment has significant positive effects on clinical outcomes in patients with knee osteoarthritis. We could also report that intermittent pneumatic compression therapy along with conventional treatment is superior to cold-pack therapy along with conventional treatment in terms of knee swelling in patients with knee osteoarthritis. This trial is registered with NCT03806322.

## 1. Introduction

Swelling is the most common symptom of knee osteoarthritis (OA), which negatively affects knee mechanics and muscle activity in patients with OA [1], indicating the need to eliminate the swelling in the early period of rehabilitation. The use of cold therapy, which is easily available and economical, for acute musculoskeletal injuries has been clearly established [2, 3]. Although cold therapy has been shown to have a statistically significant effect in improving the range of motion (ROM) and decreasing knee swelling, it is associated with safety-related concerns, such as causing skin burns and superficial nerve paralysis [4, 5]. Study results regarding the efficacy of cold therapy on clinical outcomes in patients with chronic conditions, such as knee swelling, have been inconsistent [6, 7], implicating the need for a different approach to provide better patient outcomes.

Intermittent pneumatic compression (IPC) for limb swelling is a common treatment option in patients with lymphedema and venous leg ulcers [8]. IPC is based on a passive increase of blood flow by applying external pressure to the extremity. Although IPC is being primarily used in hospitalised patients for the prevention of deep venous thrombosis (DVT) [9, 10], its efficacy for DVT prevention in outpatients has been recently demonstrated [11]. Keehan et al. reported that an IPC device used for a foot injury reduced the time to surgery and total hospital stay, thereby reducing costs for patients with ankle fractures [12]. Another study revealed that IPC was an effective, complication-free method to improve walking ability in patients with arterial claudication [13]. However, despite its widely accepted use and positive effects on circulatory conditions, the literature on the use of IPC in musculoskeletal injuries, especially OA, is limited [10].

In this study, we investigated whether IPC in combination with conventional treatment improves ROM, muscle strength, knee swelling, pain intensity, and functional status in patients with knee OA. We also compared outcomes of IPC with conventional treatment and cold-pack (CP) therapy with conventional treatment in patients with knee OA.

## 2. Materials and Methods

### 2.1. Participants

**Figure 1:**
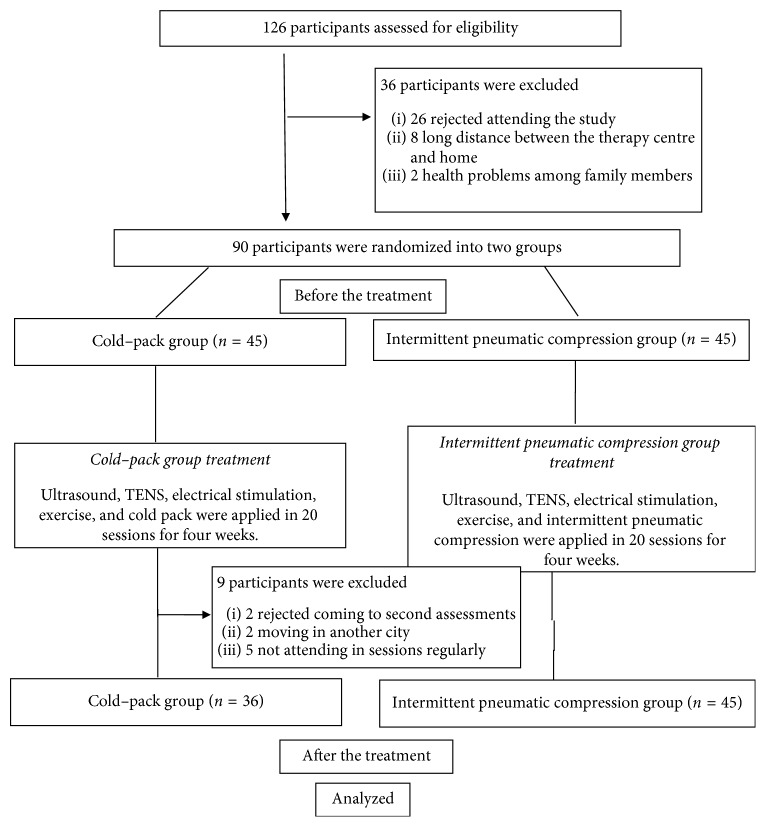
Flow chart of the study protocol and exclusion diagram for the patients.

This study was performed at the Burcu Physical Therapy Centre. A total of 126 patients were diagnosed as having knee OA according to the criteria of the American College of Rheumatology (ACR) [14] and categorised into either OA stage 2 or 3 according to Kellgren–Lawrence criteria [15]. Of the 126 patients, 36 were excluded because of failure to complete the study (declined to participate, family health problems, long distance between therapy centre and home, etc.; Figure 1), and 90 patients who met the eligibility criteria were enrolled and scheduled for follow-up visits after treatment. Because nine CP treatment group patients were lost to follow-up, the final assessment set included 36 CP group patients and 45 IPC group patients.

Exclusion criteria included previous knee surgery, malignancy, circulation disorder, conditions preventing exercise or causing muscle weakness, pregnancy, diagnosis of mental disorder, scar tissue, and metal implants.

### 2.2. Randomisation of Participants

Simple randomisation via closed-envelope technique was used to allocate the patients to either IPC or CP group. Different physiotherapists assessed patients and administered the treatment. The assessor therapist was blinded to group allocation and treatment modalities. The study was approved by the University Faculty of Medicine Ethics Committee for Clinical Research (09.2011.0061), and written informed consent was obtained from all patients before beginning the treatment.

### 2.3. Outcomes

Medical history, age, sex, height, weight, and patient history were recorded during the first evaluation. Parameters evaluated included ROM, muscle strength, knee swelling, pain intensity, and functional status.

Knee swelling was measured using a tape measure [16]. During assessment, the patient was supine with the therapist aiming to maintain the hip in a neutral position. The knee was relaxed and extended as much as possible. The therapist placed a small dot with a pen 1 cm proximal to the base of the patella using a nonelastic tape measure. The tape measure was carefully placed just above the dot, and the therapist measured knee joint circumference. The mean value of three measurements of knee circumference was used for data analysis. A universal goniometer [17] was used to measure the range of knee flexion, a digital dynamometer (J-TECH Power Track II Commander, USA) [18] was used to measure quadriceps femoris and hamstring muscle strength, and a visual analogue scale was used to measure pain intensity. The Western Ontario and McMaster Universities Osteoarthritis Index (WOMAC) [19] was used to evaluate pain, stiffness, and physical function. Following the assessments, 81 patients completed the 20-session treatment programme, which was provided 5 days a week for a total of 4 weeks [20].

The same therapist (Z. S.) measured all parameters and completed assessment before and after treatment. Final assessment for 30–45 min per participant was completed the day after the 20th session.

### 2.4. Treatment Programme

Patients were randomly divided into two groups. The CP group (*n*=36) received ultrasound (US; Intelect Legend Stim, Chattanooga Group Inc., Hixson, TN, USA), transcutaneous electrical nerve stimulation (TENS; Intelect Legend Stim, Chattanooga Group Inc., Hixson, TN, USA), neuromuscular electrical stimulation (Intelect Legend Stim, Chattanooga Group Inc., Hixson, TN, USA), exercise, and CPs. The IPC group (*n*=45) received US, TENS, neuromuscular electrical stimulation, exercise, and IPC (Doctor Life® Compression Therapy, NL).

US was adjusted to 1.5 W/cm^2^ strength using a combined electrotherapy device and was applied for 5 min around the treated knee joint in each session. Small circular movements were made in the mediolateral direction using the US head. TENS was applied for 20 min at 100 Hz frequency and 10–30 mA for 75 *μ*s to eliminate disturbance during application. Neuromuscular electrical stimulation was applied once per day for 10 min. Biphasic rectangular current at 80 Hz frequency was used by employing two electrodes placed on origin and insertion points of the vastus medialis to contract the muscle. Electrical stimulation was performed on the affected knee for 10 min in cycles of 10 s of contraction and 50 s of relaxation. The 10 s contraction time, during which the simulator was on, included a 2 s increase and a 1 s decrease. Strength of electrical current was adjusted for each patient to cause contraction in the muscle while being tolerable. A CP was applied for 15 min.

IPC treatment was applied for 30 min per day. An IPC device with four sequentially inflated chambers was adjusted to create a pressure of 45 mmHg. The duration of inflation and deflation of the device was 12 and 2.4 s, respectively. IPC used a pump to inflate and deflate a series of bladders to effectively squeeze the extremity and enhance venous return. This system required a wearable sleeve around the extremity and was connected to an external pump via a system of tubes [21].

All patients followed the same exercise programme, which was based on a study by Kuru et al. [22]. The exercise programme consisted of knee exercises, including strengthening and stretching of quadriceps and hamstring muscle, flexibility, and endurance exercises. Ten sets of each exercise were performed three times a day. Patients rested for 3 min between each exercise set.

Exercises and treatment protocol were applied by the same therapist (O. A.) for both groups.

### 2.5. Statistical Analysis

All statistical analyses were performed using IBM SPSS version 11.5 software (IBM Corporation, USA), with a *p* value of <0.05 considered statistically significant. All numerical data were expressed as mean ± standard deviation.

Shapiro–Wilk test for normality was used for all parameters. Parametric tests were used in the study because all parameters were normally distributed. In both treatment groups, data obtained from patients before and after treatment were assessed using the paired sample *t*-test. Independent sample *t*-test was used to analyse the differences between the groups.

Sample size calculation, performed using G-power version 3.1, was based on the primary outcome measure (knee joint ROM in degrees) [23]. Power and level were set to 0.85 and 0.05, respectively. *A priori* analysis for the required sample size indicated that at least 32 participants would be needed in each group. Therefore, at least 36 participants were included in each group.

## 3. Results

The study included 68 female (84%) and 13 male (16%) patients, of whom, 81 (45 in the IPC treatment group and 36 in the CP group) completed the study. There was no statistically significant difference between the groups for age, height, body weight, and body mass index (*p* > 0.05) (Table 1).

There were statistically significant improvements in the IPC group (*p* < 0.05) after the treatment in terms of knee circumference, knee flexion ROM, quadriceps and hamstring muscle strength, pain intensity, WOMAC-pain, and WOMAC-stiffness (Table 2).

In the CP group, knee flexion, quadriceps and hamstring muscle strength, pain intensity, WOMAC-pain, and WOMAC-stiffness were significantly improved after the treatment (*p* < 0.05) (Table 2).

Pre- and posttreatment circumferential measurement of the knee demonstrated no statistically significant difference in the CP group (*p* > 0.05) (Table 2).

Comparison of knee circumference values between groups revealed statistically significant differences. The IPC group had significantly better improvement in circumferential measurement of the knee than the CP group (*p* < 0.05) (Table 3).

No adverse effects following the use of IPC or CP were reported in our trial.

## 4. Discussion

This study investigated whether IPC with conventional treatment compared with CP with conventional treatment had better effects on ROM, muscle strength, knee swelling, pain intensity, and functional status. To the best of our knowledge, this is the first randomised controlled trial (RCT) to investigate the short-term effects of IPC on treatment outcomes in patients with knee OA.

Rutherford et al. [1] reported that knee swelling in patients with knee OA affects knee joint mechanics, muscle balance, and muscle activity. Palmieri-Smith et al. revealed that inhibition of quadriceps function is caused by a knee joint swelling [24]. Another study reported that joint swelling and effusion impaired the proprioceptive function in osteoarthritic knee joints [25]. Pain and functional limitation caused by swelling impair activities of daily living, inhibiting individuals with knee OA with joint pain, and limited mobility to participate in physical activities. Therefore, early-stage goals of rehabilitation should include prevention and elimination of knee swelling [26, 27].

The results of our single-blind study showed that IPC with conventional treatment decreased knee swelling better than CP with conventional treatment in patients with knee OA, confirming the advantage of IPC over CP in knee OA.

IPC with conventional treatment seems to have a significant effect on knee swelling because of circulation and blood flow regulation [28, 29]. IPC may affect circulation of lower extremities in patients with a swollen knee, which is the opposite of the local effects conferred by CP therapy. This may explain the results of this study. In addition, Praxitelis et al. reported that different types of IPC devices significantly increased the venous return [30]. In a systematic review by Philips and Gordon, it was reported that IPC for 45 min with a pressure between 30 and 60 mmHg had significant positive effects in adults and children with lymphedema [31]. Zuj et al. also demonstrated that IPC applied to the lower limb enhanced the actions of the muscle pump by increasing blood flow [32]. Maffiodo et al. concluded that using IPC for cardiocirculatory recovery was feasible in participants affected by a lower limb disease [33].

Holmström and Härdin [34] used a prospective study of 60 patients to compare the effects of integrated cold and compression (cryo/cuff) with a control group. No significant difference in swelling was shown between the groups. In another prospective study of 40 patients undergoing a total knee replacement, no difference in swelling was found between the use of cold compression and that of no-cold compression [35].

CP therapy is currently applied to reduce the degree of swelling after acute injuries [5]. Although it is one of the most frequently used therapeutic modalities to decrease swelling, the effects of CP therapy on swelling are disputed. CP therapy has an analgesic function and can relieve pain. However, no significant difference was found for the circumferential measurement of the knee after 20 sessions of CP treatment in our study.

There are various rehabilitative approaches to treat OA, but there have been no reports in the literature about the effectiveness of IPC on swelling. This is the first study to report the effectiveness of IPC on knee swelling in patients with knee OA. Based on our findings, we suggest that IPC is superior to CP in OA treatment protocol to reduce knee swelling.

We also found significant improvements in ROM, muscle strength, pain intensity, and functional status in both the IPC and CP groups, in accordance with the findings of Külcü et al. [36], who reported that conventional treatment significantly improved the functional status in patients with knee OA. Similarly, Yarahmadi et al. [37] reported that combined intervention including cold therapy effectively reduced the pain intensity.

We did not find a statistically significant difference between the two groups in terms of ROM, muscle strength, pain, or functional status. It may be surmised that an increase in knee ROM occurred because of the decrease in pain and increase in functional status following the conventional treatment programme applied to both groups.

One limitation of our study is the lack of long-term follow-up to determine the impact of IPC therapy on outcomes in patients with knee OA. In addition, we assessed knee swelling using a tape measure, which is a reliable and valid measurement technique, although a more objective evaluation would include the use of imaging techniques (US or magnetic resonance imaging, etc.). Further studies are needed to assess the long-term impact of the treatment in rheumatic conditions, including knee OA.

## 5. Conclusion

According to the findings of this study, the addition of IPC to a conventional treatment programme has better outcomes in patients with knee swelling than the addition of CP to a conventional treatment programme.

## Figures and Tables

**Table 1:** Baseline demographic and outcome characteristics of the subjects in both groups.

	IPC group (*n*=45)	Cold-pack group (*n*=36)	*p*
Age (year)	50.77 ± 9.49	52.25 ± 6.95	0.439
Gender (f/m)	37/8	31/5	—
Height (cm)	165.95 ± 8.27	164.88 ± 6.84	0.536
Body weight (kg)	79.48 ± 13.56	78.13 ± 13.81	0.660
Body mass index (kg/m^2^)	28.90 ± 5.78	28.72 ± 5.65	0.891
Knee flexion (degree)	104.8 ± 16.8	105.6 ± 15.4	0.976
Quadriceps MS (N/m)	61.6 ± 19.1	59.8 ± 8.4	0.603
Hamstring MS (N/m)	63.7 ± 15.9	58.9 ± 10.8	0.300
Knee swelling (cm)	40.8 ± 3.9	38.7 ± 2.6	0.164
Pain intensity (cm)	5.0 ± 2.1	5.4 ± 2.0	0.440
WOMAC-pain (score)	7.9 ± 4.2	7.7 ± 2.9	0.822
WOMAC-stiffness (score)	1.6 ± 1.2	1.7 ± 1.4	0.910
WOMAC-physical function (score)	20.2 ± 10.7	20.9 ± 6.6	0.751

SD: standard deviation; f: female; m: male; IPC: intermittent pneumatic compression. Values are means with standard deviation.

**Table 2:** Findings on outcome measures at baseline (before treatment) and the end of treatment (after treatment) in both groups.

Outcomes	IPC group (*n*=45)				Cold-pack group (*n*=36)			
	Before treatment	After treatment	t	*p*	Before treatment	After treatment	*T*	*P*
Knee flexion (degree)	104.8 ± 16.8	111.2 ± 16.8	−6.517	**0.001** ^*∗*^	105.6 ± 15.4	111.2 ± 13.4	−4.710	**0.001** ^*∗*^
Quadriceps MS (N/m)	61.6 ± 19.1	68.1 ± 18.5	−4.088	**0.001** ^*∗*^	59.8 ± 8.4	64.1 ± 9.6	−3.817	**0.001** ^*∗*^
Hamstring MS (N/m)	63.7 ± 15.9	67.7 ± 18	−2.806	**0.007** ^*∗*^	58.9 ± 10.8	61.5 ± 10.4	−2.278	**0.029** ^*∗*^
Knee swelling (cm)	40.8 ± 3.9	38.2 ± 3.9	4.651	**0.001** ^*∗*^	38.7 ± 2.6	38.2 ± 2.4	1.673	0.103
Pain intensity (cm)	5.0 ± 2.1	3.2 ± 2.1	6.628	**0.001** ^*∗*^	5.4 ± 2.0	3.9 ± 1.9	5.038	**0.001** ^*∗*^
WOMAC-pain (score)	7.9 ± 4.2	6 ± 3.9	5.257	**0.001** ^*∗*^	7.7 ± 2.9	5.9 ± 2.2	5.311	**0.001** ^*∗*^
WOMAC-stiffness (score)	1.6 ± 1.2	1.4 ± 1.4	1.545	0.130	1.7 ± 1.4	1.6 ± 1.2	0.473	0.639
WOMAC-physical function (score)	20.2 ± 10.7	16.3 ± 9.9	4.438	**0.001** ^*∗*^	20.9 ± 6.6	16.8 ± 6.2	5.497	**0.001** ^*∗*^

Values are means with standard deviation. Paired sample *t*-test. *p* < 0.05^*∗*^. MS: muscle strength; IPC: intermittent pneumatic compression.

**Table 3:** Mean and SD of the change in the outcome measures for the IPC and cold-pack groups.

Outcomes	IPC group (*n*=45)	Cold-pack group (*n*=36)	*t*	*p*
ΔKnee flexion (degree)	6.33 ± 6.51	6.25 ± 7.96	−0.008	0.994
ΔQuadriceps MS (N/m)	6.53 ± 10.72	4.28 ± 6.73	1.262	0.211
ΔHamstring MS (N/m)	4.02 ± 1.62	2.68 ± 1.45	1.825	0.072
ΔKnee swelling (cm)	−2.60 ± 0.86	−0.49 ± 0.92	2.243	**0.028** ^*∗*^
ΔPain intensity (cm)	−1.78 ± 1.80	−1.46 ± 1.74	−1.481	0.143
ΔWOMAC-pain (score)	−1.91 ± 2.43	−1.80 ± 2.03	0.120	0.905
ΔWOMAC-stiffness (score)	−0.28 ± 1.25	−0.11 ± 1.40	−0.716	0.476
ΔWOMAC-physical function (score)	−3.91 ± 5.91	−4.13 ± 4.51	−0.237	0.813

Values are means with standard deviation. Independent sample *t*-test. *p* < 0.05^*∗*^. SD: standard deviation; MS: muscle strength; IPC: intermittent pneumatic compression.

## Data Availability

The data used to support the findings of this study are available from the corresponding author upon request. NCT03806322 is the clinical registration number of this study.
